# *Strongyloides ratti*: In Vitro and In Vivo Activity of Tribendimidine

**DOI:** 10.1371/journal.pntd.0000136

**Published:** 2008-01-23

**Authors:** Jennifer Keiser, Kai Thiemann, Yvette Endriss, Jürg Utzinger

**Affiliations:** 1 Department of Medical Parasitology and Infection Biology, Swiss Tropical Institute, Basel, Switzerland; 2 Department of Public Health and Epidemiology, Swiss Tropical Institute, Basel, Switzerland; Case Western Reserve University School of Medicine, United States of America

## Abstract

**Background:**

Strongyloidiasis is a truly neglected tropical disease, but its public health significance is far from being negligible. At present, only a few drugs are available for the treatment and control of strongyloidiasis.

**Methodology/Principal Findings:**

We investigated the activity of tribendimidine against third-stage larvae (L_3_) of *Strongyloides ratti* in vitro and against juvenile and adult stages of the parasite in vivo. *S. ratti* larvae incubated in PBS buffer containing 10–100 µg/ml tribendimidine died within 24 hours. A single 50 mg/kg oral dose of tribendimidine administered to rats infected with 1-day-old *S. ratti* showed no effect. The same dose administered to rats harboring a 2-day-old infection showed a moderate reduction of the intestinal parasite load. Three days post-exposure a significant reduction of the immature worm burden was found. Administration of tribendimidine at doses of 50 mg/kg and above to rats harboring mature *S. ratti* resulted in a complete elimination of the larval and adult worm burden. For comparison, we also administered ivermectin at a single 0.5 mg/kg oral dose to rats infected with adult *S. ratti* and found a 90% reduction of larvae and a 100% reduction of adult worms.

**Conclusion/Significance:**

Tribendimidine exhibits activity against *S. ratti* in vitro and in vivo. The effect of tribendimidine in humans infected with *S. stercoralis* should be assessed.

## Author Summary

Although an estimated 30–100 million individuals are infected with the parasitic roundworm *Strongyloides stercoralis*, which can cause strongyloidiasis, it is a so-called neglected tropical disease. There are only very few drugs available for treating strongyloidiasis. We evaluated the strongyloicidal properties of the anthelmintic compound tribendimidine in vitro and in an animal model. Larvae from *S. ratti* incubated in PBS buffer containing 10–100 µg/ml tribendimidine died within 24 hours. Tribendimidine showed a significant activity against adult *S. ratti* harbored in rats: oral administration of tribendimidine at single doses of 50 mg/kg and above resulted in a complete elimination of larvae and adult worms. A single 50 mg/kg oral dose of tribendimidine was less effective against the migrating tissue stages, in particular the 1- and 2-day-old *S. ratti*. In view of our findings, the effect of tribendimidine against *S. stercoralis* infections in humans should be assessed.

## Introduction

An estimated 30–100 million people are infected with *Strongyloides stercoralis*, the causative agent of strongyloidiasis, and yet this is a truly neglected tropical disease [1]. One explanation is that current diagnostic tools have limitations [2]. Whilst *S. stercoralis* mainly occurs in tropical and subtropical areas, endemic foci also occur in temperate regions such as Spain or the United States [3]. Serious clinical problems have been observed in *S. stercoralis*-infected patients who are immunocompromised due to a co-infection with human T-cell leukaemia virus type 1 (HTLV-1) or HIV, or corticosteroid-treated patients [4]. However, the global burden of strongyloidiasis is currently not known. The growing evidence that an infection with *S. stercoralis* is a risk factor for biliary tract cancer needs to be considered when estimating the burden of strongyloidiasis [5].

As with other nematodes (*Ascaris lumbricoides*, hookworms and *Trichuris trichiura*) and trematodes (e.g. *Schistosoma* spp.), there are only a few drugs available for the treatment and control of strongyloidiasis [6]. Albendazole and mebendazole were found to be safe, but multiple treatment courses repeated over several weeks were required to achieve acceptable cure rates [7]. Another benzimidazole, thiabendazole, is highly efficacious (two treatment courses of 25–50 mg/kg are commonly given for 3–4 days 2 weeks apart), but severe adverse events, including liver dysfunction and neuropsychiatric symptoms, have been observed [8]. Ivermectin, a semi-synthetic macrocyclic lactone, which was developed as a veterinary anthelmintic, is safe and efficacious, and is now the drug of choice for strongyloidiasis [9]. Ivermectin resistance in humans infected with *S. stercoralis* has not been reported thus far, but host and parasite-specific resistance to the drug has been reported in veterinary medicine [9].

Tribendimidine is an aminophenyldimidine derivative of amidantel. Tribendimidine is safe and has a broad spectrum of activity against numerous nematode species, including *A. lumbricoides, Enterobius vermicularis* and the hookworms (*Ancylostoma duodenale* and *Necator americanus*) [10]. Tribendimidine has been approved by Chinese regulatory authorities in 2004 [10] and phase IV trials have been completed recently [11]. Efforts are ongoing to secure a western registration for tribendimidine, in order for the treatment to be considered for global soil-transmitted helmithiasis control usage. In our recent work, we have documented the in vivo activity of tribendimidine against a number of trematodes, namely the intestinal fluke *Echinostoma caproni* [12] and the two liver flukes *Clonorchis sinensis* and *Opisthorchis viverrini* [13].

Here, we investigated the in vitro activity of tribendimidine against third-stage larvae (L_3_) of *Strongyloides ratti*. Moreover, we evaluated the dose-response relationships of single oral doses of tribendimidine against adult *S. ratti* harbored in rats, and assessed the in vivo activity of tribendimidine against different immature stages of the parasite in the rat model.

## Materials and Methods

### Animals and parasites

All in vivo studies presented here were carried out at the Swiss Tropical Institute (Basel, Switzerland) in accordance with Swiss national animal welfare regulations (permission no. 2081).

Male Wistar rats (n = 48, age: 3 weeks, weight: ∼80 g) were purchased from RCC (Itingen, Switzerland). Rats were kept in groups of 4 in macrolon cages under environmentally-controlled conditions (temperature: ∼25°C; humidity: ∼70%; 12 h light and 12 h dark cycle) and acclimatized for 1 week.

The *S. ratti* life cycle has been maintained at our institute for 15 years by serial passage through rats. *S. ratti* L_3_ were obtained from the faeces of infected rats following standardized procedures based on the Baermann technique [14].

### Drugs

Tribendimidine was synthesized and kindly provided by the Department of Pharmaceutics, National Institute of Parasitic Diseases, Chinese Center for Disease Control and Prevention (Shanghai, China). Ivermectin was purchased from Sigma Aldrich (Buchs, Switzerland). A stock solution of tribendimidine at 10 mg/ml was prepared with 60% DMSO for the in vitro studies. For the in vivo studies, both drugs were prepared in homogenous suspensions in 7% Tween-80 and 3% ethanol before oral administration.

### In vitro studies

Freshly harvested *S. ratti* L_3_ were washed 3 times with PBS buffer and incubated in 6-well microtiter plates (Costar) containing 4 ml PBS buffer (pH 7.3). 800 *S. ratti* L_3_ were used for each control and experimental group. The worms were incubated in three serial drug dilutions of tribendimidine, i.e. 100, 10 and 1 µg/ml for up to 96 hours (h). Each experiment was carried out in triplicate and then repeated once. The control wells contained 0.06% DMSO. Cultures were kept at 25°C in an atmosphere of 5% CO_2_. Larval counts were performed immediately after exposure and at 1, 2, 24, 48, 72 and 96 h post-exposure under a dissecting microscope.

### In vivo studies

Rats were infected subcutaneously with 1300 freshly harvested *S. ratti* L_3_. To analyse the effect of tribendimidine on adult *S. ratti*, 5 days post-exposure, 5 groups with 4 rats each were administered tribendimidine at doses of 200, 100, 50, 25 and 12.5 mg/kg, respectively (first set of experiments: doses 50–200 mg/kg; second set of experiments: doses 12.5 and 25 mg/kg). Ivermectin was given to 4 rats at a single 0.5 mg/kg oral dose. Eight rats were left untreated and served as controls.

Stool samples from all rats investigated were collected shortly before treatment and daily for 4 days after treatment. From each treatment group 250 mg stool was homogenized with 2.5 ml PBS buffer and 10 aliquots of 10 µl stool suspension were analyzed for the presence of rhabditiform larvae. The average rhabditiform larvae count per gram of stool was calculated.

Seven days post-treatment rats were euthanised by CO_2_. At necropsy the intestine was removed from the pylorus to the ileocecal valve, placed in a Petri dish containing 10 ml of PBS buffer (pH 7.3) opened longitudinally and incubated for 3 h. Ten aliquots of 10 µl PBS were analyzed for the presence of rhabditiform larvae and adults and the average worm counts were recorded.

To determine whether tribendimidine has an effect on the tissue stages of *S. ratti*, 12 rats were treated intragastrically, either on day 1, 2 or 3 post-exposure (4 rats each), with a single 50 mg/kg oral dose of tribendimidine. One group with 4 infected but untreated rats served as control. Stool samples were collected between days 5 and 9 post-exposure and processed as described above. Rats were sacrificed on day 9 post-exposure and the intestine was analysed for the presence of *S. ratti* adults and rhabditiform larvae as explained before.

### Statistical analysis

Statistical analyses were done with version 2.4.5 of Statsdirect statistical software (Statsdirect Ltd; Cheshire, UK). The effect of tribendimidine was assessed by comparing the mean number of *S. ratti* rhabditiform larvae in the stool and *S. ratti* adults and rhabditiform larvae in the intestine in the treatment group with the mean number of larvae and adults in the respective control group. The responses between the medians of the treatment and control groups regarding larvae and stool and larvae and adults in the intestines were analysed with the Kruskal-Wallis (KW) test. Differences in medians were considered to be significant at a significance level of 0.05.

## Results

### In vitro findings

Table 1 summarizes the effect of *S. ratti* L_3_ after exposure to tribendimidine at different concentrations in vitro. *S. ratti* exposed to tribendimidine at 10 or 100 µg/ml contracted immediately and had a coiled shape appearance. The worms died within 24 h. No effect was observed with the lowest concentration (1 µg/ml) of tribendimidine: 66.6% of *S. ratti* were still active after an incubation period of 72 h. Ninety-six h post-incubation 58.4% of *S. ratti* showed no movement when exposed to this concentration, similar to the control group, where 60.0% of the worms were found to be inactive.

**Table 1:** Observed immobility of *S. ratti* L_3_ after exposure in vitro to tribendimidine at three different concentrations.

Drug	Drug concentration (µg/ml)	% of worms inactive (SD) after incubation for						
		5 min	1 h	2 h	24 h	48 h	72 h	96 h
Control	No treatment	40.0 (28.5)	55.0 (7.1)	36.7 (20.1)	23.4 (23.2)	26.7 (16.5)	50.0 (28.6)	60.0 (14.1)
Tribendimidine	1	30.0 (26.7)	0 (0)	33.4 (2.4)	23.4 (6.3)	5.0 (7.1)	33.4 (9.4)	58.4 (17.0)
	10	30.0 (28.6)	48.3 (6.2)	70.0 (32.4)	100 (0)	-	-	-
	100	36.6 (35.6)	98.4 (2.4)	100 (0)	-	-	-	-

SD: standard deviation.

### Effect of tribendimidine on adult *S. ratti*: dose-response relationship

**Figure 1:**
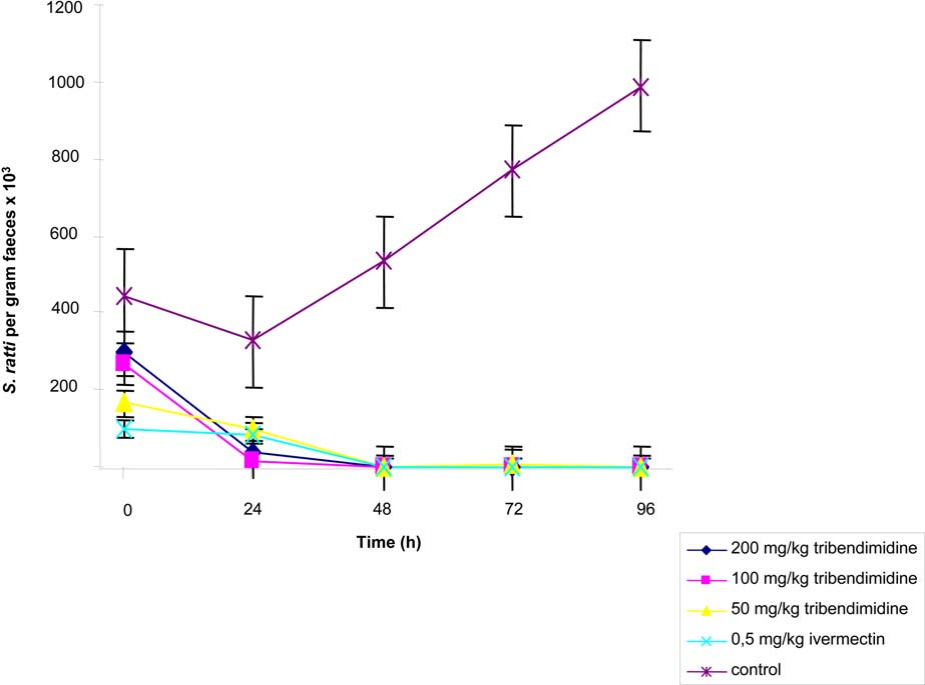
Mean (SE) fecal larvae output of 4 *S. ratti*-infected rats that remained untreated and 4 *S. ratti*-infected rats that were treated on day 5 post-exposure with either 0.5 mg/kg ivermectin, or either 50, 100 or 200 mg/kg tribendimidine.

**Figure 2:**
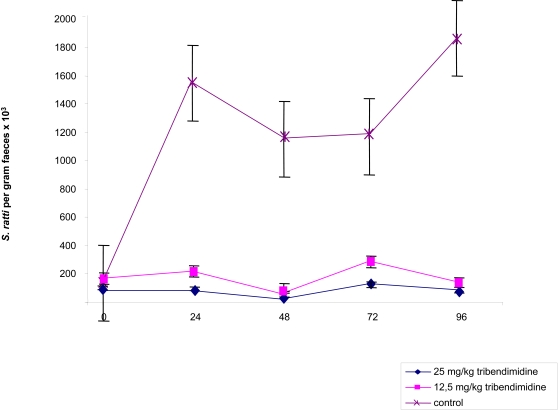
Mean (SE) fecal larvae output of 4 *S. ratti*-infected rats that remained untreated and *S. ratti*-infected rats treated on day 5 post-exposure with either 12.5 or 25 mg/kg tribendimidine.

Figure 1 shows the effect of tribendimidine on *S. ratti* rhabditiform larvae harvested from rat fecal samples as assessed by quantitative stool examination. In the first set of experiment (evaluating single 50, 100 and 200 mg/kg oral doses of tribendimidine) on 5 daily stool examinations, between 3300 (6 days post-exposure) and 10,000 (9 days post-exposure) rhabditiform larvae per gram of stool were estimated in the group of untreated control animals. No larvae were found in fecal samples obtained from rats treated with single 100 or 200 mg/kg oral doses of tribendimidine and 0.5 mg/kg ivermectin commencing 48 h post-treatment. While no larvae were found at 48 h and 96 h post-treatment with 50 mg/kg tribendimidine, a low mean of 100 larvae per gram of stool was estimated 72 h post- treatment (Figure 1). Untreated rats in the second set of experiment (assessing the activity of 12.5 and 25 mg/kg tribendimidine) passed between 1000 and 18,600 *S. ratti* rhabditiform larvae per gram of stool on days 5 to 9 post-exposure. Larvae were also present in the stools of rats treated with 25 and 12.5 mg/kg tribendimidine; the highest numbers of rhabditiform larvae were detected 72 h post-treatment, namely 1300 and 2900 larvae per gram of stool in rats treated with 25 and 12.5 mg/kg, respectively (Figure 2). However, treatment had a significant effect on larvae presence in stools (KW = 67.1, degree of freedom (df) = 2; *P* < 0.001).

The effect of tribendimidine against *S. ratti* rhabditiform larvae and adults in the intestine, as assessed by worm burden reduction, is summarized in Table 2. The untreated control rats harbored a mean of 1012 *S. ratti* rhabditiform larvae and 413 adult worms in their intestines. Tribendimidine given at doses of 50, 100 and 200 mg/kg resulted in complete elimination of larvae and adult worms.

**Table 2:** Effect of ivermectin and tribendimidine (different doses) against adult *S. ratti* harbored in rats.

Treatment	Dose (mg/kg)	No. of rats cured*/investigated	Mean larval burden (SD)	Mean adult worm burden (SD)	Total larval burden reduction (%)	KW	*P*-value	Total adult worm burden reduction (%)	KW	*P*-value
Control 1^a^	No treatment	0/4	1012 (640)	413 (284)	-	-	-	-	-	-
Control 2^b^	No treatment	0/4	2025 (1001)	750 (387)	-	-	-	-	-	-
Tribendimidine	12.5^b^	0/4	925 (846)	125 (96)	54.3	6.83	0.041	83.3	9.46	0.008
	25^b^	0/4	175 (150)	0	91.4			100		
	50^a^	4/4	0	0	100	14.61	0.002	100	10.28	0.016
	100^a^	4/4	0	0	100			100		
	200^a^	4/4	0	0	100			100		
Ivermectin	2.5^a^	4/4	150 (150)	0	90	4.58	0.032	100	4.0	0.045

*: number of rats without *S. ratti.*

a first experiment.

b second experiment.

KW: Kruskal-Wallis.

SD: standard deviation.

For comparison, ivermectin administered at 0.5 mg/kg achieved a significant larval reduction (90.0%; KW = 4.58, *P* = 0.032) and a complete elimination of adult worms.

The second control group harbored 750 *S. ratti* adults and 2025 larvae in their intestines. Tribendimidine at 25 mg/kg produced a 91.4% reduction of *S. ratti* larvae and a complete reduction of adult worms. When tribendimidine was given at 12.5 mg/kg, the adult worm burden was reduced by 83.3% and the larval burden by 54.4%. There was a significant difference in the larval (KW = 6.83, df = 2, *P* = 0.041) and the adult worm (KW = 9.46, df = 2, *P* = 0.009) burden between these 2 treatments and the control groups.

### Effect of tribendimidine on juvenile *S. ratti*

**Figure 3:**
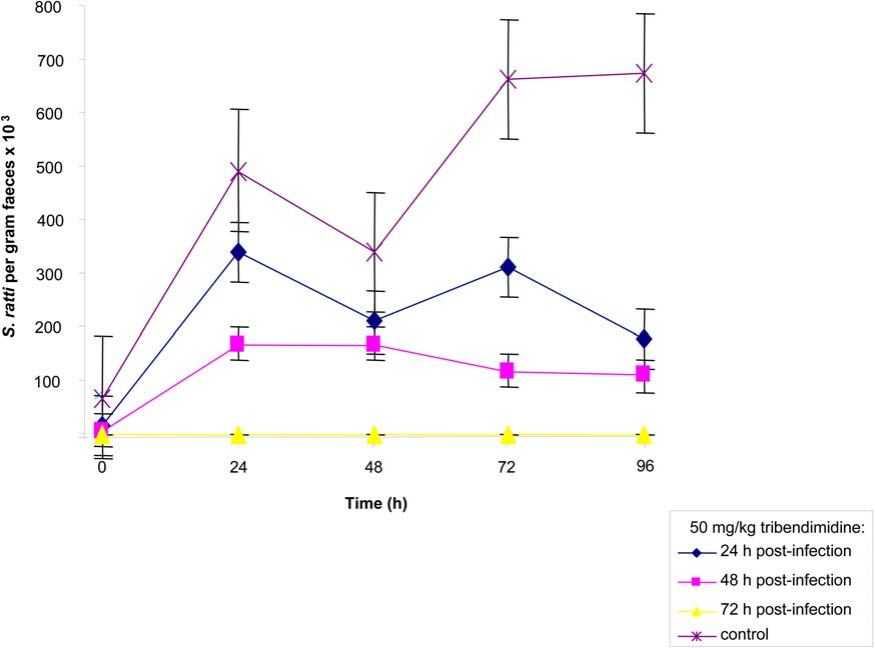
Mean (SE) fecal larvae output of 4 *S. ratti*-infected rats that remained untreated and *S. ratti-*infected rats treated with 50 mg/kg tribendimidine either on day 1, 2 or 3 post-exposure.

Figure 3 shows the effect of tribendimidine given 1–3 days post-exposure on *S. ratti* rhabditiform larvae present in stool. The number of rhabditiform larvae in the control group increased from 700 on day 5 post-exposure to 6700 rhabditiform larvae per gram of stool 4 days later. A larval reduction ranging from 30.6% (6 days post-exposure) to 73.1% (9 days post-exposure) was observed in fecal samples of rats treated with 50 mg/kg tribendimidine on day 1 post-exposure. In rats treated with tribendimidine at 50 mg/kg on day 2 post-exposure, a reduction of rhabditiform larvae ranging between 50.0% (7 days post-exposure) and 83.6% (9 days post-exposure) was observed. Finally, no rhabditiform larvae were found in 5 consecutive stool samples from rats treated with 50 mg/kg of tribendimidine on day 3 post-exposure. Administration of tribendimidine to rats harboring tissue stages of *S. ratti* had a significant effect on the presence of larvae in stool (KW = 14.1; df = 3, *P* = 0.002).

Table 3 summarizes observed larvae and adult worm burden reductions in the intestines of rats following treatment with tribendimidine. Administration of a single 50 mg/kg oral dose of tribendimidine on day 1 post-exposure to *S. ratti*-infected rats showed no effect on the intestinal larval and adult parasite load. Treatment of infected rats 48 h post-exposure with a single 50 mg/kg oral dose of tribendimidine resulted in larvae and adult worm burden reductions of 41.0–61.5%. Finally, when tribendimidine (50 mg/kg) was given 72 h post-exposure, a 98.9% reduction of larvae in the intestines was observed. There was a significant difference between the number of larvae (KW = 9.65, *P* = 0.021) and adult worms (KW = 6.29, *P* = 0.098) recovered from the intestines of treated and non-treated control rats.

**Table 3:** Effect of a single 50 mg/kg oral dose of tribendimidine against immature stages of *S. ratti* harbored in rats.

Treatment	Treatment day post-exposure (stage)	No. of rats cured*/investigated	Mean larval burden (SD)	Mean adult worm burden (SD)	Total larval burden reduction (%)	Total adult worm burden reduction (%)
Control	No treatment	0/4	2375 (1190)	150 (58)	-	-
Tribendimidine	1 (L_3_ larvae in lungs or in cranial or nasal cavities)^a^	0/4	2800 (2546)	225 (263)	0	0
	2 (some preadolescent stages in intestine)^a^	0/4	1400 (716)	50 (58)	41.1	61.5
	3 (some preadolescent stages in intestine)^a^	0/4	25 (50)	50 (58)	98.9	61.5

*: number of rats without *S. ratti.*

a Harder et al. (2001) [25].

Kruskal-Wallis (KW) testing difference in total larval burden reduction (KW = 9.66, df = 3, *P* = 0.021) and adult worm burden reduction (KW = 6.29, df = 3, *P* = 0.098).

SD: standard deviation.

## Discussion

Discovered in the mid-1980s [15], detailed laboratory investigations and subsequent clinical testing for safety and efficacy have led to tribendimidine being registered in China, in early 2004, as an anthelmintic drug with a broad spectrum of activity. In experimentally-infected animals, tribendimidine showed excellent activity against major nematode infections, i.e. *A. lumbricoides* and the hookworms, particularly *N. americanus* [10]. Tribendimidine has also shown excellent activity against a number of trematodes, e.g. *C. sinensis*, *E. caproni* and *O. viverrini* in different rodent models [12],[13]. Here, we extended in vitro and in vivo activity testing of tribendimidine to yet another helminth, namely *S. ratti*, which is a commonly used experimental model of a nematode infection [16]. A single 50 mg/kg oral dose of tribendimidine administered to rats harboring adult *S. ratti* resulted in complete worm burden reductions. The same dose given to rats infected with juvenile *S. ratti* revealed a significant reduction of the larval burden already 3 days post-exposure. However, no effect was found when tribendimidine was administered 1 day post-exposure and only a moderate effect was observed at day 2 post-exposure. Whether immature *S. ratti* are less sensitive to tribendimidine or whether drug levels are lower in the tissues where larvae reside at this age remains to be investigated. Further studies are necessary to investigate the effect of higher and also multiple doses of tribendimidine against immature *S. ratti*.

It is interesting to compare our results with a 2-day treatment regimen of ivermectin, the current drug of choice for strongyloidiasis. Two doses of 0.3 mg/kg ivermectin resulted in complete worm burden reduction of adult *S. ratti* in mice [17]. A 2-day dose of 0.5 mg/kg ivermectin was highly efficacious against the migrating and lung stages of this parasite, resulting in worm burden reductions of 91–96% [18]. In our own experiment, a single 0.5 mg/kg oral dose of ivermectin resulted in a 90% larval reduction and a complete elimination of adult worms. On the other hand, albendazole or thiabendazole were found to be less efficacious against immature *S. ratti* even when multiple doses were administered. For example, a 3-day treatment schedule of 50 mg/kg albendazole and thiabendazole administered on days 4–6 post-exposure cured rats infected with adult *S. ratti.* However, no or only low cure rates (up to 33%) were observed when these drugs were administered during the lung and tissue stages of the parasite on days 1, 2 and 3 post-exposure [19]. Interestingly, 2 doses of 50 mg/kg mebendazole were found to be highly efficacious against the tissue stages of *S. ratti* [20].

Our in vitro studies revealed that worms incubated in the presence of 100 µg/ml tribendimidine died within 2 h. The worms had a coiled appearance. It has been suggested that tribendimidine, which, similar to amidantel is biotransformed into p-(1-dimethlyamino ethylimino) aniline (S.H. Xiao, pers. comm.), acts as agonist at the level of the acetylcholine receptor [21]. The rapid onset of action of tribendimidine is supported by our in vivo studies since no larvae were found in stools of rats treated with at least 50 mg/kg of tribendimidine 48 h post-treatment. A previous investigation showed that tribendimidine acted similarly rapidly when it was administered to mice infected with the intestinal trematode *E. caproni.* Scanning electron microscopic investigations revealed that severe damage of the tegument already occurred 2 h after drug administration and 8 h post-treatment the majority of worms had been expelled [12].

Concluding, we have documented in vitro and in vivo activities of tribendimidine against *S. ratti*. Our findings warrant further investigations, which is justified as follows. First, discovery and development of novel anthelmintic drugs in general [22],[23] and strongylocidal drugs in particular, is limited. Hence, over the past decade only a few compounds have been examined in the *S. ratti*-rat model [24],[25]. Second, tribendimidine has recently been registered in China as an anthelmintic drug, and it might thus be deployed as an additional control tool against major helminth infections [26]. Efforts are ongoing to pursue registration of tribendimidine in a 1^st^ tiered regulatory agency so that the drug could eventually be integrated in global helminth control programs. Since many helminth infections show large geographical overlaps, it will be important to monitor the effect of tribendimidine on concomitant infections of different nematodes and trematodes. We are currently in the process of examining the effect of tribendimidine against *S. stercoralis* in people co-infected with this parasitic roundworm and other nematodes and trematodes.
